# Web-based intervention to improve quality of life in late stage bipolar disorder (ORBIT): randomised controlled trial protocol

**DOI:** 10.1186/s12888-018-1805-9

**Published:** 2018-07-13

**Authors:** Kathryn Fletcher, Fiona Foley, Neil Thomas, Erin Michalak, Lesley Berk, Michael Berk, Steve Bowe, Sue Cotton, Lidia Engel, Sheri L. Johnson, Steven Jones, Michael Kyrios, Sara Lapsley, Cathrine Mihalopoulos, Tania Perich, Greg Murray

**Affiliations:** 1 Centre for Mental Health, Swinburne University of Technology, Melbourne, Australia; 2 Department of Psychiatry, University of British Columbia, Vancouver, Canada; 3 IMPACT Strategic Research Centre, School of Medicine, Deakin University Barwon Health, Geelong, Australia; 4 Department of Psychiatry, University of Melbourne, Melbourne, Australia; 5 Orygen, the National Centre of Excellence in Youth Mental Health, Parkville, Australia; 6 Centre for Youth Mental Health, University of Melbourne, Melbourne, Australia; 7 Florey Institute for Neuroscience and Mental Health, Melbourne, Australia; 8 Deakin University, Geelong, Australia; 9 University of California, Berkeley, CA USA; 10 Spectrum Centre for Mental Health Research, Faculty of Health and Medicine, Lancaster University, Lancaster, UK; 11 Flinders University, Adelaide, Australia; 12 Western Sydney University, Sydney, Australia; 13 School of Psychiatry, University of New South Wales, Sydney, Australia; 14 Centre for Mental Health, Swinburne University of Technology, PO Box 218, Hawthorn, VIC 3122 Australia

**Keywords:** Randomised controlled trial (RCT), Bipolar disorder, Web-based intervention, Mindfulness, Psychoeducation, Quality of life, Stage, Depression, Mania

## Abstract

**Background:**

The primary objective of this randomised controlled trial (RCT) is to establish the effectiveness of a novel online quality of life (QoL) intervention tailored for people with late stage (≥ 10 episodes) bipolar disorder (BD) compared with psychoeducation. Relative to early stage individuals, this late stage group may not benefit as much from existing psychosocial treatments. The intervention is a guided self-help, mindfulness based intervention (MBI) developed in consultation with consumers, designed specifically for web-based delivery, with email coaching support.

**Methods/design:**

This international RCT will involve a comparison of the effectiveness and cost-effectiveness of two 5-week adjunctive online self-management interventions: Mindfulness for Bipolar 2.0 and an active control (Psychoeducation for Bipolar). A total of 300 participants will be recruited primarily via social media channels. Main inclusion criteria are: a diagnosis of BD (confirmed via a phone-administered structured diagnostic interview), no current mood episode, history of 10 or more mood episodes, no current psychotic features or active suicidality, under the care of a medical practitioner. Block randomisation will be used for allocation to the interventions, and participants will retain access to the program for 6 months. Evaluations will be conducted at pre- and post- treatment, and at 3- and 6- months follow-up. The primary outcome measure will be the Brief Quality of Life in Bipolar Disorder Scale (Brief QoL.BD), collected immediately post-intervention at 5 weeks (T1). Secondary measures include BD-related symptoms (mania, depression, anxiety, stress), time to first relapse, functioning, sleep quality, social rhythm stability and resource use. Measurements will be collected online and via telephone assessments at baseline (T0), 5 weeks (T1), three months (T2) and six months (T3). Candidate moderators (diagnosis, anxiety or substance comorbidities, demographics and current treatments) will be investigated as will putative therapeutic mechanisms including mindfulness, emotion regulation and self-compassion. A cost-effectiveness analysis will be conducted. Acceptability and any unwanted events (including adverse treatment reactions) will be documented and explored.

**Discussion:**

This definitive trial will test the effectiveness and cost-effectiveness of a novel QoL focused, mindfulness based, online guided self-help intervention for late stage BD, and investigate its putative mechanisms of therapeutic action.

**Trial registration:**

ClinicalTrials.gov: NCT03197974. Registered 23 June 2017.

**Electronic supplementary material:**

The online version of this article (10.1186/s12888-018-1805-9) contains supplementary material, which is available to authorized users.

## Background

Bipolar disorder (BD) is a serious mental disorder affecting approximately 4% of the adult population [1]. Large prospective and cross-sectional studies suggest that about 50% of people diagnosed with BD can be considered ‘late stage’ (experiencing ≥10 mood episodes) [2, 3]. This ‘late stage’ group carry a disproportionate burden of functional impairment and chronic depressive symptoms [3], double the risk of relapse, and significantly impaired functioning and QoL [3]. Stage of illness impacts prognosis, course outcome and treatment needs. There is evidence that responsivity to pharmacotherapy varies by stage of illness, and there is similar evidence for some psychotherapies [4].

### Adjunctive psychological treatment for late stage BD

Current adjunctive psychological treatments for BD emphasise relapse-prevention by monitoring triggers and avoiding stress. However, this may be detrimental to self-esteem in late stage BD where relapse is often unrelated to discernible life events [5]. Indeed, having experienced more than 12 episodes of BD has been found to predict a *negative* response to cognitive behaviour therapy (CBT) [6]. In late stage BD, therefore, symptom-focused treatments may be less beneficial than approaches that recognise the unavoidability of suffering, emphasise redefinition of life goals, and prioritise QoL outcomes [7, 8]. These priorities are consistent with the “third wave” [9] or contextual cognitive-behavioural therapies [10], which typically prioritise *mindfulness* - an awareness of present experience and a non-judgemental stance towards that experience [11].

Empirical research into mindfulness based interventions (MBIs) for BD has primarily explored face-to-face delivery, with Mindfulness Based Cognitive Therapy (MBCT) [12] the primary target of investigation. Studies report benefits for BD symptoms and associated psychological variables, in particular anxiety and emotion regulation [13]. Beneficial effects of MBIs in other domains have been reported including QoL [14], well-being, stress and rumination [15]. Evidence for effects on relapse prevention is limited: one study found no benefits for bipolar relapse [16], while another found decreased depressive relapse in a bipolar subset of people with recurrent depression [17]. A recent meta-analysis on the efficacy of MBIs as an adjunctive treatment for BD showed significantly beneficial effects on depressive and anxiety symptoms in within-group analysis [18]. However, this significance was not observed in comparison with control groups, limiting conclusions due to the small number of controlled studies and small sample sizes. Furthermore, studies examining mindfulness as part of another treatment modality (e.g., Acceptance and Commitment Therapy; ACT) were excluded from their review, as were short-duration (< 3 week) interventions and self-help interventions delivered online [8]. A further limitation of studies to date is their focus on symptom/distress reduction, which is at odds with the main goals of CCBT approaches [19]. Mechanisms of action of such approaches are still unclear, as process variables (e.g., mindfulness, acceptance, self-compassion) have not been examined in mediational analyses. In summary, further clinical trials are needed to determine the efficacy of MBIs for improving QoL in BD and understanding their mechanisms of action.

### Maximising access to adjunctive psychological treatments

Web-based psychological interventions have great potential to complement treatment as usual and overcome barriers to accessing psychological assistance for BD (e.g., cost, time, trust in professionals) [20]. Such interventions have demonstrated short- and long-term benefits for a range of mental disorders [21] and are acceptable to people with BD [22, 23]. There is a lack of consistent evidence regarding effectiveness of web-based psychological interventions for BD as most trials to date have focused on feasibility. Recent findings confirm that MBIs are effectively delivered via the web [24], including for mood disorder populations [25]. The benefits of low intensity web-based interventions are yet to be widely disseminated to people with BD, highlighting difficulties in translation into practice.

### Development and piloting of the current intervention

Our international team of researchers, clinicians and consumers developed and piloted a novel, guided self-help web-based psychological intervention to improve QoL in late stage BD [8]. The low-intensity MBI is specifically tailored for late stage BD, drawing from Acceptance and Commitment Therapy (ACT), Mindfulness Based Cognitive Therapy (MBCT) and Compassion-focused Therapy (CFT). The intervention was originally named ORBIT (Online, Recovery-oriented, Bipolar Individualised Tool), but to keep participants unaware of the present trial’s hypothesised superior condition, ORBIT now refers to the project as a whole. For this report, the active intervention is titled ‘Mindfulness for Bipolar 2.0’ and the active control condition is titled ‘Psychoeducation for Bipolar’, but: to minimise expectancy effects, the names and key features of each arm are not mentioned in recruitment or consent processes.

Published pilot data suggests the first iteration of Mindfulness for Bipolar was feasible, safe and potentially effective in improving QoL [8]. No further published studies have investigated web-based MBIs specifically tailored for late stage BD. However, in related severe and chronic mental illness populations, there is support for the efficacy of generic MBIs and MBCT [26, 27], as well as ACT [28, 29]. Evidence suggests that those with chronic mental illness benefit from mindfulness strategies to mitigate the distressing effects of symptoms (via mindful acceptance of internal experiences), and improve self-concept (via promoting an experience of self as observer, moderating self-evaluations and encouraging commitment to valued goals despite symptoms) [30]. Indeed, there is evidence that those with a significant BD history may specifically benefit from a bipolar-tailored version of MBCT [15, 31, 32].

### The present project

Building on pilot study findings, we now describe the development of and protocol for a definitive RCT of the second iteration of the MBI (Mindfulness for Bipolar 2.0) for improving QoL in late stage BD.

The overarching aim of the present RCT is to assess the effectiveness and cost-effectiveness of a novel, web-based intervention in improving QoL in late stage BD vs. an active control.

#### The trial’s primary hypothesis

Relative to an active control (Psychoeducation for Bipolar), it is predicted that Mindfulness for Bipolar 2.0 will significantly improve QoL (baseline to endpoint change) on the Brief QoL.BD [33] immediately post-intervention. Data will additionally be collected at three and six months post-baseline, allowing examination of the trajectory of intervention effects over time.

#### Secondary hypotheses

It is anticipated that relative to Psychoeducation, the Mindfulness intervention will improve QoL across the full 6 months of the trial. A significant time X group assignment effect is therefore predicted. It is further anticipated that, relative to Psychoeducation, the Mindfulness condition will be associated with improvements in self-rated anxiety, self-rated depressive syndrome, observer rated depression, and attrition rates. Possible group differences will also be explored in a range of secondary outcomes for which directional predictions are not made: observer-rated manic symptoms; functional impairment; functionally oriented measure of QoL; sleep quality, social rhythm stability, and episode relapse (time to first). It is also anticipated that the Mindfulness condition will be more cost-effective than Psychoeducation. Potential moderators and mediators of treatment effects will also be explored as described below.

## Method

This trial is conducted by an international multidisciplinary team of researchers and clinicians in Australia, Canada, USA and the UK. The study was reviewed and approved by Swinburne University of Technology Human Research Ethics Committee (2016/289). Trial objectives and protocol align with all aspects of Good Clinical Practice [34], the WHO Trial Registration Data Set (Version 1.2.1) and Standard Protocol Items: Recommendations for Interventional Trials (Additional file 1) guidelines [35]. The trial has been registered with ClinicalTrials.gov (NCT03197974). Any changes to this trial protocol will be described in this trial registry. Findings will be reported to the scientific community according to Consolidated Standards of Reporting Trials (CONSORT) eHEALTH criteria [36].

### Trial design

The trial is a prospective, parallel group, rater-blind, superiority RCT with a one-to-one allocation ratio comparing the Mindfulness for Bipolar 2.0 intervention with a structurally equivalent, validated and therapeutically credible active control condition (Psychoeducation for Bipolar). The primary endpoint is immediately post-treatment (upon completion of the 5-week intervention). Follow-up time points are at 3-months and 6-months. Both interventions are guided self-help, and not intended to replace treatment as usual. The study setting is online, and participation in both arms occurs through a secure server.

### Randomisation

Participants will be sequentially allocated to intervention arms using a one-to-one ratio by predetermined permuted block randomisation with a block size of 10. The permutation sequence within blocks will be randomly generated by SAS Version 9.4, overseen by an off-site statistician, and coded into the website so randomisation is automated and free of potential allocation bias. Participants are unaware of the primary hypothesis of the study, and have no prior knowledge of the two intervention types, but are unavoidably aware of the intervention to which they have been randomised. To reduce expectancy effects for superiority of the intervention over the control condition, the trial is framed as a comparison between two interventions designed to improve QoL. Assessors will remain unaware of treatment condition, and assessors who become aware of the treatment condition will, if possible, be replaced. Success in keeping assessors unaware of the treatment condition will be quantified by asking interviewers to guess allocation upon completion of the RCT, and assessing whether they can do so at greater than chance levels. Statistical analyses will be conducted blind to treatment allocation by an off-site statistician.

### Recruitment and assessment

The study will be administered and conducted through a single-site, with recruitment primarily via open social media sites (e.g., International Bipolar Foundation Facebook site). Recruitment will also be facilitated in four English-speaking countries via advertisements disseminated online through public and mental health sites and social media, listservs and traditional advertisements (offline) posted in clinical services.

**Fig. 1:**
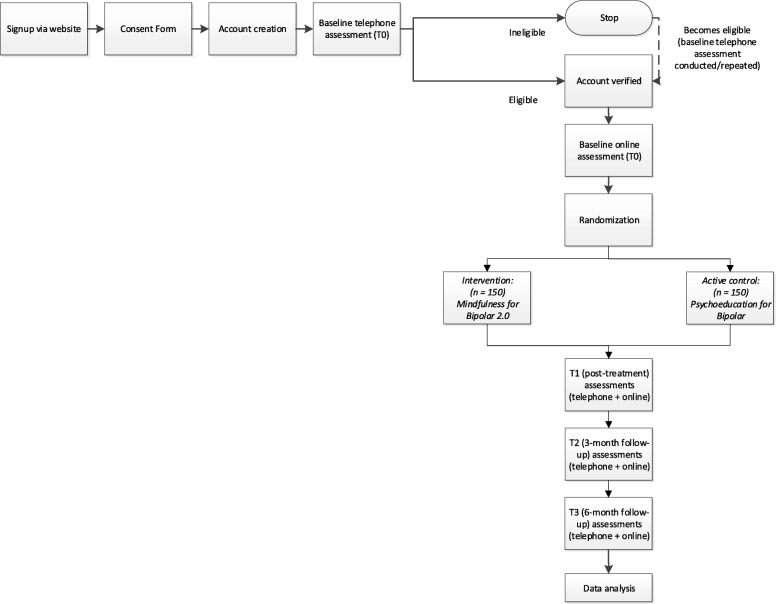
Participant flow diagram

As summarised in the flow diagram (see Fig. 1), individuals interested in participating will be invited to visit the study website (https://www.orbitonline.org/) where they can sign up, view and provide their consent to the participant information and consent form, provide their contact details (including details of their medical health professional), and respond to initial screening questions regarding eligibility (i.e., received a diagnosis of BD from a mental health professional, experienced 10 or more mood episodes).

An assessor from the research team will then contact potential participants via telephone to conduct the first component of the baseline assessment (T0) to confirm eligibility criteria and assess comorbidities. Potential participants deemed eligible except for current episode status/psychotic symptoms/active suicidality (see exclusion criteria below) will be offered a later assessment (one month following initial phone call) to determine eligibility at that time. Eligible participants will complete the second component of the baseline assessment (online questionnaires) (T0), followed by randomisation. An online coach will be assigned to the participant (contact initiated via a ‘welcome’ message). Participants will be asked to complete post-intervention (5-weeks, T1), 3-month (T2) and 6-month (T3) post-baseline assessments, involving a telephone interview and online questionnaires (see Table 1 for schedule of assessments). Participation in each assessment (phone and online components) will be compensated with USD$25 gift vouchers.

**Table 1:** Schedule of assessments

Measure	Modality	T0 baseline	T1 (5 weeks post-baseline)	T2 (3 months post-baseline)	T3 (6 months post-baseline)
MINI Neuropsychiatric Interview (including Columbia Suicide Severity Rating Scale, C-SSRS)	Telephone	X	X	X	X
Brief Quality of Life in Bipolar Disorder (QoL.BD)	Online	X	X	X	X
Montgomery-Asberg Depression Rating Scale (MADRS)	Telephone	X	X	X	X
Young Mania Rating Scale (YMRS)	Telephone	X	X	X	X
Quick Inventory of Depressive Symptoms-Self Report (QIDS-SR)	Online	X	X	X	X
DASS-21 (anxiety and stress sub-scales)	Online	X	X	X	X
Functioning Assessment Short Test (FAST)	Telephone	X	X	X	X
Pittsburgh Sleep Quality Index (PSQI)	Online	X	X	X	X
Sleep, Circadian Rhythms and Mood questionnaire (SCRAM)	Online	X	X	X	X
Resource Use Questionnaire (RUQ)	Online	X		X	X
Assessment of Quality of Life-Eight Dimension (AQoL-8D)	Online	X	X	X	X
Five Facet Mindfulness Questionnaire (FFMQ)	Online	X	X	X	X
Self-Compassion Scale (SCS)	Online	X	X	X	X
Difficulties in Emotion Regulation Scale-16 item (DERS-16)	Online	X	X	X	X
Ruminative Response Scale-10 item (RRS-10)	Online	X	X	X	X
Responses to Positive Affect scale (RPA)	Online	X	X	X	X
Nonattachment to Self Scale	Online	X	X	X	X
Depressive Experience Questionnaire-Self Criticism Six-item Scale (DEQ-SC-6)	Online	X	X	X	X
Short Form of the Revised Almost Perfect Scale (SAPS)	Online	X	X	X	X
Medication adherence	Online	X	X	X	X
User feedback and engagement	Online and telephone		X	X	X
Reasons for withdrawal	Telephone		X	X	X

Assessors (masters-level in psychology) will be trained on study measures. Inter-rater reliability will be established for key observer-rated outcome measures (i.e. Montgomery-Asberg Depression Rating Scale, MADRS; Young Mania Rating Scale, YMRS), telephone assessment interviews will be recorded (except for participants who decline), and inter-rater checks will be conducted every three months. If rater drift is identified, assessors will be retrained.

### Strategies to maximise data quality

Online questions will be made mandatory where possible to reduce missing data. Comprehensive data cleaning (range and other distributional checks, comparison with published norms where relevant) will be conducted prior to analyses.

### Inclusion criteria

To ensure ready translation, minimally restrictive inclusion/exclusion criteria have been set. Inclusion criteria are as follows:

1. Aged 18–65 years
2. Received a diagnosis of BD from a mental health professional (GP or Psychiatrist)
3. Confirmation of DSM-IV diagnosis of BD as assessed by telephone interview with the M.I.N.I. International Neuropsychiatric Interview (MINI) [37]
4. experienced 10 or more episodes of mania, hypomania or depression (assessed within the context of the MINI and assessor prompts, e.g., life events)
5. under the care of a medical practitioner (at least one contact within the past 12 months) and able to provide contact details of local emergency service
6. sufficient understanding of written and spoken English to provide informed consent and engage with the intervention
7. ready daily access to the internet and adequate internet literacy

### Exclusion criteria

Exclusion criteria are as follows:

1. experiencing a current episode of depression, hypomania or mania (assessed by MINI)
2. currently psychotic (assessed by MINI)
3. active suicidal ideation (assessed by Columbia Suicide Severity Rating Scale, C-SSRS) [38]

### Risk management

Risk management procedures have been developed through our experience with other online interventions and websites for BD [22, 39–43] and through consultation with the *CREST.BD* Community Advisory Group. Procedures pay particular attention to suicide risk, distinguish between solicited and spontaneously reported events, and are reviewed weekly during the trial by the study’s executive committee. To minimise impact of any adverse events, being under the care of a medical practitioner is an inclusion criterion, and participants consent to this professional potentially being contacted by the researchers as part of a ‘red flag decision tree’ (detailed below). The informed consent statement explains that the medical practitioner or local emergency department remains the participant’s first point of contact. Both the pre-registration page and informed consent statement highlight participants’ central role in their own safety and wellbeing, emphasising that the website does not act as an emergency service, and that the website is not monitored in real time. Links to emergency resources will be provided on the website (e.g., *unsuicide.**wikispaces.com*).

Participants will be withdrawn from the trial if their participation compromises clinical care as determined by the study’s executive committee. On a case-by-case basis, this might include hospital admission, acute hypomania or mania, psychotic symptoms or active suicidal intent.

Adverse events will be monitored via questions at post-test and both follow-up time points, and any adverse events incidentally reported through participant contact with coaches, forum posts, or assessments will also be recorded. Severity of any adverse events, and causal relationship to the trial will be assessed through participant self-report and logged. Adverse events will be reviewed at monthly executive meetings and 6-monthly meetings of the independent trial committee (who will also explore patterns of adverse events by condition). Serious adverse events suspected or known to be related to participation in the trial will be reported to Swinburne University of Technology Human Research Ethics Committee.

Clinical risks emerging will be addressed through a detailed ‘red flag decision tree’ which captures the various points at which the researchers could become aware of risk information (particularly suicidality), namely, baseline and follow-up (post-intervention, 3-month, 6-month) assessment interviews, posted comments on the website (e.g., forum content) and communications with the online coach (see below). Red flag events are those where there is immediate risk of harm (e.g., scoring high on suicide items, forum posts or coach emails suggesting active suicidality), as contrasted with other concerns highly prevalent in this population (e.g., comments about waxing and waning symptoms, connectedness or distress). Only in the former case have participants consented for researchers to break confidentiality and communicate with their clinician or emergency services, and these events are considered red flags for action of some kind. The decision tree distinguishes between red flag information suggesting immediate risk of harm for which real-time intervention is feasible/recommended (e.g., when active suicidality is identified during a phone assessment), and when it is not (e.g., when the research team becomes aware of active suicidality mentioned in a forum post from 48 h previous). All trial staff will be comprehensively trained on protocol procedures.

### Intervention content

To minimise attrition and non-adherence the intervention is brief, with a 5-week structured participation time, with each new module released sequentially. Participants will complete modules at their own pace: content within modules is organised into ‘chunks’ allowing exploration in briefer or longer individual sessions, depending on personal preferences. Suggested guidelines on using the program to maximise benefit will be provided, including spending a minimum of 1–2 h each week on content and ongoing skills-practice (with daily practice recommended for mindfulness skills and mood monitoring).

Both interventions comprise four modules, with new module content delivered sequentially each week over four weeks. The fifth week is positioned as an opportunity to consolidate skill development. Participants can return to previous modules across the program at any time. To support ongoing generalisation of skills, participants retain access to the program for the 6-month follow-up period.

To maximise engagement, the interventions follow best practice in persuasive system design. Features known to impact engagement are included: (1) *dialogue support* (praise from coach and forum moderator, email reminders), (2) *social support* (social facilitation through discussion threads in moderated forums) and (3) *primary task support* (best practice principles for modularisation of content, personalisation/monitoring of progress, prompted self-monitoring, and rehearsal) [44]. Website presenters include experienced clinical academics and consumers with lived experience of BD. Topics within modules are organised primarily around videos (2–3 min in length), followed by text, reflective exercises, audio exercises and additional information PDFs. Participants can comment publically on videos (via comments feed) and complete exercises which are private but can be shared with others users of the website.

Coaching support improves adherence to web interventions [40]. Both interventions therefore include personal coaching support through asynchronous email contact with a trained online coach. Participants can send as many messages as they like to their coach, and will receive one response per week. Completed activities can be shared with the coach to facilitate skill development. A moderated forum is included to develop a sense of online community. The forum is moderated by trained consumers with lived experience of BD, who will also seed discussion threads with encouraging tips.

#### Mindfulness for bipolar 2.0

This is a bespoke web-based intervention tightly targeted at QoL outcomes in late stage BD. Intervention content is framed around the development of five overlapping skills: Mindfulness (self-awareness, mindfulness as a tool for emotion regulation), Values and Committed Action (identifying personal values as a guide to action), Acceptance (of negative experiences, contrasted with struggling), Defusion (creating distance from unproductive thoughts, emotions and sensations), and Self-compassion (cultivating self-compassion in the context of ongoing symptoms and previous disappointments).

Content draws from best-practice psychological interventions of potential utility in BD, specifically ACT, MBCT and CFT [45]. To support generalisation to real world settings and maintenance upon completion of the 5-week active phase, classical principles of behaviour change (as commonly used, for example, in Cognitive Behavioural Therapy) are applied. Website aesthetics, structure and navigation are informed by our previous experience, particularly development of a web-based intervention for people with serious mental disorder [46].

Intervention content is well suited to web delivery, using multi-media tools (video, audio, downloadable information sheets, etc.) to introduce skills that participants can practice experientially with well-planned exercises. Real-world application of skills is emphasised throughout, and facilitated through online and downloadable goal-setting and monitoring tools.

#### Psychoeducation for bipolar

Psychoeducation is a meaningful comparator given that it is beneficial as an adjunctive treatment for BD and is readily translated into web format [47]. The active control condition here, Psychoeducation for Bipolar, is a 5-week, web-based guided self-help intervention informed by the approach of Colom and Vieta [48]. Material is organised into four modules (*Bipolar and You, Treatments, Knowing the Signs,* and *Staying Well*), with a focus on factual information about BD including available medications and treatments, skill development (mood monitoring, recognising triggers and early warning signs) and strategies to stay well (lifestyle factors, coping behaviours, crisis planning).

### Outcome measurements

The schedule of assessments is provided in Table 1. Assessments are performed as close as practicable to specified time points. All clinician-rated assessments are conducted by telephone by expertly trained assessors, unaware of treatment allocation. All self-report assessments are completed online via a secure, encrypted online survey platform (Qualtrics). Participants are contacted via email to arrange a time for phone assessments and to prompt completion of online assessments. If an email response is not received within two days, an SMS reminder is sent to the participant. Assessors will monitor the completion of questionnaires on a regular basis and make telephone calls to facilitate completion where such support may be required. The MINI is conducted via telephone at baseline to confirm eligibility, establish comorbid diagnoses and record demographic variables.

#### Primary outcome measure

The primary outcome, QoL, will be measured at T1 using the total score of the Brief QoL.BD [33]. This measure was developed specifically for repeated measures use, assessing 12 factor-analytically derived domains (Physical, Sleep, Mood, Cognition, Leisure, Social, Spirituality, Finance, Household, Self-esteem, Independence, and Identity) from the parent instrument (the 56-item Qol.BD). The parent instrument has demonstrated strong effect size associations with objective measures of functioning [49], generic QoL measures [33], and BD-linked cognitive processes [50]. In the validation sample (*N* = 224 patients), Cronbach’s α for the 12 items of the Brief QoL.BD was 0.87, and standard deviation of total scores was 8.76, giving a reliable change on the Brief QoL.BD of 3.16 points [33]. Sensitivity analysis found the 12-item Brief QoL.BD had superior sensitivity to change in clinician-rated symptoms than commonly used generic QoL measures. Brief QoL.BD scores have been shown to be sensitive to intervention effects in two published RCTs [22, 51], and the measure has been validated for online use.

#### Secondary outcome measures

The following measures will be used to assess secondary outcomes. Each measure will be administered at T0, T1, T2 and T3 (see Table 1).

1. Montgomery-Asberg Depression Rating Scale (MADRS) [52]. A gold standard, psychometrically sound interviewer-rated scale to assess depression symptoms.
2. Young Mania Rating Scale (YMRS) [53]. A gold standard, psychometrically sound interviewer-rated scale to assess manic symptoms.
3. Quick Inventory of Depressive Symptomatology-Self-Report (QIDS-SR) [54]. A self-report measure of depression symptoms with high internal consistency (*α* = 0.87), that correlates highly with established clinician-rated scales including the Hamilton Rating Scale of Depression (*r* = 0.86) [54].
4. Depression Anxiety Stress Scale (DASS-21) [55]. A valid and reliable self-report measure of depression, anxiety and stress (total scores and the latter two sub-scales will be used).
5. Functioning Assessment Short Test (FAST) [56]. An interviewer-rated measure evaluating functional impairment across six different domains (autonomy, occupational functioning, cognitive functioning, financial issues, interpersonal relationships, leisure time), validated in patients with BD. The measure has excellent test-retest reliability and internal consistency (*α* = 0.95) [57, 58].
6. Pittsburgh Sleep Quality Index (PSQI) [59]. One of the most widely used self-report measures of sleep quality. The components and items have demonstrated strong internal consistency; global and component scores have been shown to be stable over time [59].
7. Sleep, Circadian Rhythms and Mood questionnaire (SCRAM) [60]. A self-report measure to assess overlap between sleep, circadian rhythms and mood. Preliminary psychometric analyses indicate adequate test-retest reliability and good internal consistency (*α* = 0.80).
8. Relapse. The MINI [37] will be conducted at each assessment to determine illness episodes. The Time to relapse measure (TIME) [61] assesses relapse or time to intervention, with intervention defined as initiation, discontinuation, or dose adjustment of a treatment, initiation of psychotherapy or electroconvulsive therapy, visit to an emergency provider or hospitalisation in response to new mood symptoms.

#### Other outcome measures and mechanism variables

The following measures will be administered at T0, T1, T2 and T3 (see Table 1), with the exception of the Resource Use Questionnaire (administered at T0, T2 and T3 in order to standardise timeframe assessment for economic analyses).

1. Resource Use Questionnaire (RUQ, unpublished). Although differences in service use are not formally hypothesised as an outcome of the intervention, the self-reported RUQ assesses for any differences in mental health service utilisation between groups before, during, and following the intervention. To derive costing for the economic analysis, this study will use a purpose designed RUQ, which is largely based on previous RUQs used by the study team in other mental health economic evaluations. The RUQ was modified for the international context of the study, assessing direct health care costs (including out-of-pocket costs) and productivity costs. RUQ information will be supplemented by Australian Government Medicare data and Pharmaceutical Benefits Schedule for Australian participants.
2. Assessment of Quality of Life-Eight Dimension (AQoL-8D) [62]. A self-report measure to assess health-related QoL for use in economic evaluations that has been comprehensively validated and demonstrated high test-retest reliability [63] .
3. Five Facet Mindfulness Questionnaire (FFMQ) [64]. A self-report measure of the dispositional tendency to be mindful in daily life. The 39-item measure has satisfactory internal consistency and demonstrates good sensitivity to change [65–67].
4. Self-Compassion Scale (SCS) [68]. A self-report measure assessing the degree of self-compassionate responding towards oneself during hard times. A recent examination of the 26-item measure indicated it is a psychometrically valid and theoretically coherent measure of self-compassion [69] . Excellent internal consistency (*α* = 0.89) for the total score was demonstrated in a bipolar sample [70].
5. Difficulties in Emotion Regulation Scale-16 item (DERS-16) [71]. A self-report measure to assess overall emotion regulation difficulties. The measure has excellent internal consistency (*α* = 0.92) and good test-retest reliability [71].
6. Ruminative Response Scale (RRS-10) [72]. A self-report measure of tendency to ruminate, with adequate internal consistency and test-retest reliability.
7. Responses to Positive Affect Scale (RPA) [73]. A self-report measure to assess rumination and dampening regulation strategies. Tests of convergent validity support associations between sub-scales and mood measures, and acceptable internal consistency was established in a bipolar sample [74].
8. Nonattachment to Self Scale (Whitehead et al.: Letting go of self: nonattachment-to-self and its relationship to depression, anxiety and stress. Submitted). A self-report measure to assess the degree of dispassion/non-attachment to self-concept. The initial validation study (unpublished) of the scale demonstrated good internal consistency (*α* = 0.86), and robust correlations with other constructs in expected directions (e.g., mindfulness for convergent validity; depersonalization for discriminant validity).
9. Depressive Experience Questionnaire Self-Criticism Six-Item Scale (DEQ-SC6) [75]. A self-report measure to assess trait self-criticism, with adequate internal consistency (up to *α* = 0.84) and expected associations with pertinent constructs (e.g., emotional distress).
10. Short revised Almost Perfect Scale (SAPS) [76]. A self-report measure to assess perfectionism, with adequate internal consistency (up to *α* = 0.87). Convergent and discriminant validity was demonstrated with other indicators of perfectionism; as was criterion-validity in terms of associations with other constructs (e.g., conscientiousness, neuroticism, emotion regulation and depression).
11. Adherence to Medication. A self-report question with five response options regarding degree of adherence to medication over the past month.
12. User feedback and engagement. Participants are invited to provide feedback about the program immediately post-treatment. Usage of the site, and frequency/duration of application of skills/knowledge is assessed via self-report at the three major assessment time points (see Table 1). Upon completion of the post-intervention assessments, a subset of participants from each arm will be selected to participate in a recorded qualitative phone interview (with an investigator who is independent of study assessments) regarding their engagement with, and experience of the intervention.
13. Program usage. This is tracked automatically by the website, and data will be used to develop an algorithm operationalising use/adoption, which combines activity completion and active engagements with the intervention [77].
14. Reasons for discontinuation from study. Participants requesting to discontinue their participation will be asked (via a brief phone call from the trial coordinator) for main reasons for discontinuation, with any adverse events documented.

### Statistical analysis plan

Sample size was determined by power analysis using G*Power 3. Analyses were conducted on the primary endpoint (Brief QoL.BD score) at T1 relative to baseline. Based on our pilot study (intent-to-treat *d* = 0.52), and Brief QoL.BD change in comparable RCTs [22, 51], a between-group effect size of *d* = 0.4 was conservatively estimated. This small-to-moderate effect size is comparable to effect sizes found for adjunctive CBT on a range of outcomes in BD [78], and therefore can be considered a clinically important difference given the new intervention is low intensity, low cost, and high access. A sample size of 200 (100 in Mindfulness for Bipolar 2.0 and 100 in Psychoeducation for Bipolar) would provide at least 80% power (1-*β*) to detect an effect of this size at α = 0.05 (two-tailed). Attrition at immediate follow-up was conservatively estimated at 33% based on attrition rates in the pilot study and our earlier RCT of a web-based intervention for BD [39], so *N* = 300 randomised participants are required to generate the required sample size of 200 for the primary analysis.

#### Statistical analysis of primary outcome

Changes from baseline to immediate post-test in QoL.BD scores are hypothesised to be significantly greater in the Mindfulness versus Psychoeducation group. Statistical analyses will be conducted in accordance with the International Conference on Harmonization E9 statistical principles. All treatment-related effects will be estimated using intention-to-treat: all randomised participants will be analysed. We will attempt to follow and assess all participants regardless of level of usage of the site during the intervention period (with the exception of those explicitly discontinuing, or being withdrawn on ethical grounds).

#### Sensitivity analysis of primary outcome

In order to corroborate the robustness of the primary outcome, the following sensitivity analyses will be performed:

- Per protocol analysis: estimation of the treatment effects in those participants who actually receive the intervention. For analytic purposes, these analyses will be restricted to participants who complied with the treatment protocol, defined as (i) completed all assessments required for the analysis, and (ii) received a minimal dose of the intervention
- Intention to treat multivariate regression analysis with imputation, and adjusting for the baseline variables used to impute missing data. For subjects with missing data at either time- point, scores will be imputed using multiple imputation with 20 resamples, and imputing baseline variables age, gender, diagnosis, number of episodes, depression symptoms, country of residence and any relevant data collected post-randomisation (specific analytic method will depend on patterns of missingness in the data). Missing data will be imputed using predictive mean matching, using the 10 nearest neighbours.
- As in (b) above, but using only observed data.

#### Time trend analysis of QoL

To investigate the secondary outcome of expected superiority of Mindfulness for QoL outcomes across the full 6 months of the trial, an intention to treat mixed model analysis using T0, T1, T2 and T3 QoL.BD scores will be undertaken.

#### Secondary outcome measures

Treatment-related change in secondary outcomes analyses will be identical to those described above for the primary outcome. It is anticipated that relative to Psychoeducation, the Mindfulness condition will be associated with improvement in three outcomes: (a) self-rated state anxiety (DASS-Stress, DASS-Anxiety), (b) self-rated depression (QIDS-SR) and (c) clinician-rated depression (MADRS). It is also anticipated that relative to Psychoeducation, the Mindfulness condition will be associated with decreased dropout attrition.

Analysis of treatment-related change in remaining secondary outcomes will be exploratory. Time to first relapse will be analysed using multivariate survival analysis [79].

#### Mechanism analyses

A series of parallel multiple mediation analyses will be conducted to examine the effects of putative treatment mechanisms on QoL [80, 81]. It is anticipated that therapeutic effects of the Mindfulness intervention on QoL will be mediated by improvements in mindfulness, self-compassion and emotion regulation. Exploratory analyses will be undertaken with regards to nonattachment to self, self-criticism and perfectionism. It is anticipated that the therapeutic effects of the Psychoeducation intervention on symptoms of mania and depression, and relapses into mania and depression will be mediated by (i) self-reported adherence to medication, and (ii) biological rhythm stabilisation (SCRAM).

#### Moderators of treatment effects

Although no baseline moderators are predicted to explain significant variance in outcome (and consequently stratification is not used in allocation), a signal across standard baseline variables (age, gender, diagnosis, number of episodes, depression symptoms, and country of residence), and the putative mediating variables (above) will be explored. Potential confounds of medication use/changes and use of structured psychological interventions during follow-up will be tracked.

#### Economic analyses

A cost-effectiveness and a cost-utility analysis will be carried out alongside the ORBIT trial from both a societal perspective and a health sector perspective. Evaluation will first measure and value any change to the use of health care resources over the period of the study between the two interventions, and then compare any additional costs to the outcomes achieved. Standardised economic evaluation techniques will be used including incremental analysis of mean differences and bootstrapping to determine confidence intervals. Quality-of-adjusted life years (QALYs) will be derived from the AQoL-8D that will form a cost-utility analysis and the Qol.BD will be used for the cost-effectiveness analysis. Costs considered in this economic analysis include: (i) intervention costs, (ii) direct health care costs (including out-of-pocket costs), and (iii) productivity costs (absenteeism, presenteeism and unpaid work) [82, 83]. As it is anticipated that most participants will be recruited in Australia, a one-country costing approach will be applied using cost estimates from Australia [84]. Sensitivity analyses will be used to determine the impact of important study parameters (such as unit cost price variation). Australian participants will be asked for consent to provide access to routine data on use of health care services through Medicare (Health Insurance Commission) and the Pharmaceutical Benefits Schedule. Depending on the results, modelling may also be used to extrapolate beyond the trial time horizon.

## Discussion

This study constitutes a definitive investigation of a novel intervention aimed specifically at improving QoL in late stage BD. Consistent with Mental Health Research Network good practice guidelines and the principles of consumer engagement in research and treatment development [85, 86], the intervention has been developed in partnership with individuals with lived experience of BD.

The intervention exemplifies a significant shift in psychosocial approaches to BD. It is one of the few to investigate stage of illness-based approaches to psychotherapy, MBIs for BD, and the first to adopt a web-based platform for offering MBIs to this population. The intervention is innovative in targeting the consumer-focused priority of improving QoL; offering tailored content for late stage BD including use of lived experience material to complement third-wave psychological approaches; and adopting features of persuasive system design to maximise adherence and engagement. Examination of mechanisms of change associated with this MBI will elucidate putative treatment targets, informing both the use of this intervention as well as refining future interventions for BD.

The trial will contribute a rigorously evaluated intervention to the growing literature on low-intensity web-based self-management programs for BD.

Several limitations are noteworthy. First, while the intervention is accessible via multiple platforms (computer, tablet, smartphone), it does not currently support technical integration with mobile health (mHealth) or popular wearable technologies (e.g., actigraphy devices), which have strong potential for objective monitoring in BD [87, 88]. These restrictions arise from both resource limitations and the study’s scientific aim of tracking engagement through the website itself. Future versions of the intervention will seek to integrate such technologies. Second, the intervention has been specifically designed for late stage BD. The concept of ‘late stage BD’ remains poorly characterised, and many questions remain about illness progression and staging in BD [89]. Here, we employ the face-valid operationalisation of number of episodes (assessed by prompted self-report), while recognising that more theoretical and empirical work is required into the concept itself. Should study findings support intervention effectiveness, future RCTs will be required to determine whether findings generalise to early and mid-stage BD.

There is room for innovation in the BD psychotherapy space. Outcomes of this RCT of a low intensity web-based MBI QoL intervention tailored for late stage BD will be of great interest to researchers and end-users in light of its multiple innovations. It will contribute to tailoring therapies to individual needs and profiles, in particular stage of illness. We hope that positive findings of the RCT will support immediate scaling up of the intervention as a new tool for improving outcomes for people with the latter stage BD.

### Trial status

Recruitment is ongoing. It is anticipated that the trial will be completed (T3) by September 2019.

## Additional file

Additional file 1: SPIRIT 2013 Checklist: Recommended items to address in a clinical trial protocol and related documents. (DOC 136 kb)

## Abbreviations

ACT: Acceptance and Commitment Therapy
AQoL-8D: Assessment of Quality of Life-Eight Dimension
BD: Bipolar disorder
Brief QoL.BD: Brief Quality of Life in Bipolar Disorder Scale
CCBT: Contextual cognitive-behavioural therapies
CFT: Compassion-focused Therapy
C-SSRS: Columbia Suicide Severity Rating Scale
DASS-21: Depression Anxiety Stress Scale
DEQ-SC-6: Depressive Experience Questionnaire-Self Criticism Six-item Scale
DERS-16: Difficulties in Emotion Regulation Scale-16 item
FAST: Functioning Assessment Short Test
FFMQ: Five Facet Mindfulness Questionnaire
MADRS: Montgomery-Asberg Depression Scale
MBCT: Mindfulness Based Cognitive Therapy
MBIs: Mindfulness-based interventions
MINI: M.I.N.I. International Neuropsychiatric Interview
ORBIT: Online Recovery-oriented Bipolar Individual Tool
PSQI: Pittsburgh Sleep Quality Index
QIDS-SR: Quick Inventory of Depressive Symptomatology-Self-Report
QoL: Quality of life
QUALY: Quality-of-adjusted life years
RCT: Randomised controlled trial
RPA: Responses to Positive Affect Scale
RRS-10: Ruminative Response Scale
RUQ: Resource Use Questionnaire
SAPS: Short revised Almost Perfect Scale
SCRAM: Sleep, Circadian Rhythms and Mood questionnaire
SCS: Self-Compassion Scale
YMRS: Young Mania Rating Scale
